# Routinely Used and Emerging Diagnostic and Immunotherapeutic Approaches for Wheat Allergy

**DOI:** 10.3390/biomedicines12071549

**Published:** 2024-07-12

**Authors:** Wanqi Zheng, Christine Yee Yan Wai, Jason Ka Chun Sit, Nam Sze Cheng, Christy Wing Man Leung, Ting Fan Leung

**Affiliations:** 1 Department of Paediatrics, The Chinese University of Hong Kong, Shatin, Hong Kong; wq_zheng@outlook.com (W.Z.); christineyywai@cuhk.edu.hk (C.Y.Y.W.); jasonsit@cuhk.edu.hk (J.K.C.S.); nancycheng@cuhk.edu.hk (N.S.C.); 2 Hong Kong Hub of Paediatric Excellence, The Chinese University of Hong Kong, Shatin, Hong Kong; 3 Faculty of Medicine, The Chinese University of Hong Kong, Shatin, Hong Kong; lwmchristy@gmail.com

**Keywords:** component-resolved diagnosis, desensitization, immunology, oral immunotherapy, wheat allergy

## Abstract

Wheat, a component of the staple diet globally, is a common food allergen in children. The symptoms of wheat allergy (WA) range from skin rash to shortness of breath, significantly impairing quality of life. Following initial clinical suspicion, individuals may undergo routinely used allergy tests such as a wheat allergen-specific skin prick test (SPT), a blood test for specific immunoglobulin E (sIgE) levels, or oral food challenge. Conventional management of WA lies in wheat avoidance, yet accidental consumption may be inevitable owing to the ubiquity of wheat in various food products. This article aims to provide an overview of the immunologic pathway of WA, followed by its emerging diagnostic methods, namely alcohol-soluble SPT extracts, component-resolved diagnosis, and the basophil activation test (BAT). The mechanisms underlying wheat allergen-specific oral immunotherapy (OIT) as well as a summary of the efficacy, tolerability, and safety of related clinical trials will then be discussed.

## 1. Introduction

Wheat (*Triticum aestivum*) plays an important role in the global human diet due to its nutritional value and adaptability to diverse climatic conditions. However, the prevalence of wheat allergy (WA) poses significant concerns. Wheat has been identified as one of the top five triggers of allergic reactions in children. In Germany and Japan, it ranks as the third most common food allergen after milk and eggs [1]; according to Leung et al. and Yu et al., WA is the top cause of anaphylaxis in Thailand, Japan, and Korea [2,3]. A European meta-analysis of food allergy prevalence revealed that the overall lifetime prevalence of self-reported WA was around 1.6% (95% confidence interval [CI] 0.9–2.3) with the highest prevalence being among preschool children aged 2–5 years, while the point prevalence of food challenge-verified WA was 0.1% (95% CI 0.01–0.2) [4]. The lifetime prevalence for self-reported WA was lower than that for cow’s milk (5.7%, 4.4–6.9) and egg (2.4%, 1.8–3.0) and comparable to the respective figures for peanut allergy (1.5%, 1.0–2.1) and fish allergy (1.4%, 0.8–2.0) [4].

Wheat allergy is an immune-mediated adverse reaction triggered by the consumption of wheat or wheat-based products [5]. It includes three main types: immunoglobulin E (IgE)-mediated, non-IgE-mediated, and mixed-IgE-mediated forms [3]. The latter two types are less common, with their mechanisms yet to be delineated, and are thus not detailed herein. IgE-mediated WA involves two stages, namely sensitization and elicitation. Sensitization occurs upon initial exposure to wheat, leading to the production of wheat protein-specific IgE antibodies. During the elicitation phase, subsequent exposure triggers the interaction between cell-bound IgE and the same wheat proteins, resulting in the release of allergic mediators from mast cells and basophils. These processes lead to a range of clinical manifestations within minutes to hours after wheat consumption, including erythema, pruritus, gastrointestinal reactions, oropharyngeal symptoms, urticaria, angioedema, atopic dermatitis, rhinitis, asthma; in severe cases, WA may escalate to life-threatening anaphylaxis [6]. So far, the gold standard for immediate WA diagnosis remains oral food challenges, but these carry substantial risks to the patient and must be conducted under rigorous medical supervision [6]. In addition, the skin prick test (SPT) and allergen-specific IgE (sIgE) measurements using wheat extract suffer from low specificity and are hence not suitable for definitive WA diagnosis [5]. Therefore, other more accurate and safer methods for WA diagnosis need to be explored.

Current treatment for WA primarily relies on strict avoidance of wheat and prompt management of adverse reactions resulting from accidental exposure [7]. However, challenges arise since wheat is widely used to manufacture various food products, increasing the likelihood of inadvertent exposure. Therefore, alternative desensitization treatments are needed. Immunotherapy, comprising oral immunotherapy (OIT), sublingual immunotherapy (SLIT), and epicutaneous immunotherapy (EPIT), holds promise in this regard [7]; in particular, OIT has received more research attention for WA, although it is still in the early stages of research. Oral immunotherapy aims to induce oral tolerance to allergenic foods by modulating both innate and adaptive immune mechanisms [3,8,9], reflected by reduced secretion of inflammatory mediators from mast cells and basophils, increased levels of wheat allergen-specific immunoglobulin G 4 (IgG4), and wheat allergen-specific IgE levels that initially increase and then decrease [9].

To date, wheat allergen-specific OIT is yet to be incorporated into clinical practice due to it being in the early stages of research. Nonetheless, this article will review the immunologic pathways of WA and the mechanism of the wheat allergen-specific OIT correspondingly, on a molecular level. Integrative diagnostic methods with both routine and emerging allergy tests as well as treatment options will also be introduced, with a specific focus on oral immunotherapy.

## 2. Immune and Epigenetic Mechanism

### 2.1. Sensitization

**Figure 1:**
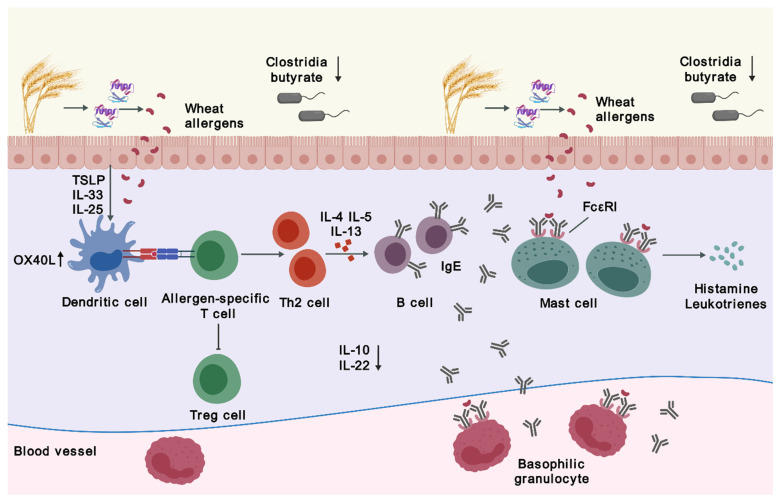
Pathophysiology of wheat allergy. Abbreviations: FcεRI, high-affinity IgE receptors; IgE, immunoglobulin E; IL, interleukin; OX40L, a ligand for tumor necrosis factor receptor superfamily member 4; Th2, T helper 2; Treg, regulatory T; TSLP, thymic stromal lymphopoietin. The figure was created with gdp.renlab.cn.

The process of IgE-mediated WA involves two stages—sensitization upon initial exposure to wheat allergens and elicitation when encountering re-exposure. Sensitization occurs when wheat allergens enter the body following digestion and absorption in the gastrointestinal tract. Epithelial-derived inflammatory cytokines, such as interleukin (IL)-25, IL-33, and thymic stromal lymphopoietin (TSLP), act on dendritic cells (DCs) and other cells to skew the immune response [8,10,11]. The tumor necrosis factor receptor superfamily member 4 (OX40) ligand (OX40L), upregulated on DCs, induces differentiation of naive T cells into T helper 2 (Th2) cells instead of regulatory T (Treg) cells, which elicit tolerogenic responses [12]. This Th2-biased immune dysregulation results in the production of IL-4, IL-5, and IL-13, promoting class switching of B cells to produce more wheat allergen-specific IgE. These IgE antibodies bind to mast cells and basophils via their high-affinity IgE receptors (FcεRI) [13,14,15,16]. Upon re-exposure to wheat allergens, allergen-antibody binding triggers IgE crosslinking and hence the activation of tissue-residing mast cells and blood-circulating basophils, swiftly releasing inflammatory and vasoactive mediators such as histamine, platelet-activating factor, and leukotrienes to induce local or systemic allergic reactions [6,16,17,18,19] (Figure 1).

### 2.2. Desensitization via Immunotherapy

Immunotherapy desensitizes the immune systems of individuals by modulating the humoral immune response and activities of T cells and DCs [3,7,8]. In immunotherapy, IgE levels rise transiently and then fall. The allergen-specific IgG levels, especially specific IgG4 (sIgG4), however, steadily increase [20,21,22], likely due to steady release from the upstream IL-10 and allergen-specific regulatory B (Breg) cell pathways. The IgG isotypes also dampen IgE-mediated responses by binding to FcγRIIb and suppressing IgE-mediated activation of basophils and mast cells [3,23,24,25]. Allergen-specific immunoglobulin A (IgA) supports antigen exclusion and enhances the specificity of desensitization [8].

Repeated allergen exposure during the OIT also induces apoptosis and anergy of allergen-specific Th2 cells alongside further activation of allergen-induced Treg cell functions. As mentioned above, Treg cells are tolerogenic, hindering mast cell activation directly via OX40-OX40L interaction and inhibiting DCs required for effector T cell activation [3,26]; clinically, they protect individuals from anaphylaxis. The synergistic effects of immunoglobulins and T cells facilitate allergen desensitization in immunotherapy. Further studies, however, are required to observe any wheat allergen-specific mechanisms of desensitization in wheat immunotherapy.

### 2.3. Epigenetics 

Currently, the evolving pattern of food allergies is attributed to complex gene–environment interactions [27,28,29]. Environmental factors influencing the susceptibility and development of food allergies are mediated through epigenetic mechanisms, including DNA methylation, non-coding RNA, and histone modifications. These mechanisms involve heritable changes in gene expression related to immune signaling pathways without altering the underlying DNA sequence [27,28,29]. Studies have shown that tolerance acquisition in children with IgE-mediated cow’s milk allergy is characterized by distinct DNA methylation patterns in Th1 and Th2 cytokine genes (such as *IL-4* and *IL-5*) and epigenetic regulation of the Treg transcription factor *FOXP3* [30,31]. Other studies have revealed different DNA methylation profiles in Th1 and Th2 cytokines when comparing non-allergic and peanut-allergic patients [32,33]. These findings suggest a link between epigenetic regulation of the immune system and food allergies. 

Environmental factors such as delivery mode, antibiotic use, and diet may trigger microbial dysbiosis and influence immunity through epigenetic mechanisms [8,34,35,36]. The gut microbiota plays a crucial role in modulating allergic responses to food antigens by activating DCs on the gut mucosal surface. These activated DCs produce IL-10 and IL-22 to stimulate the maturation of naive T cells into Treg cells and enhance the production of anti-microbial and food allergen-specific IgA, establishing food tolerance. Conversely, an imbalance of gut microbiota in the intestinal epithelium may lead to elevated levels of IL-4, IL-33, and allergen-specific IgE, driving Th2 cell-biased responses and stimulating basophil activation in the intestine [36,37,38,39]. 

Studies have shown alterations in gut microbiota in children with WA compared to those without [40]. A study by Kanchongkittiphon et al. revealed statistically significant enrichment of *Anaerostripes*, *Erysipelatoclostridium*, *Prevotella 2*, *Ruminiclostridium 5*, and *Clostridium innocuum* species in children with physician-diagnosed WA [41]. The specific bacterial groups or species involved in the induction of Treg cells in the intestinal mucosa remain unknown. It appears that genera such as *Lactobacillus*, *Bifidobacterium*, and *Clostridium*, along with microbial fermentation products like butyrate, stimulate Treg cells and alleviate food allergy symptoms [36,42,43]. So far, the evidence of the impact of dietary nutrition and the microbiome on the epigenetic modulation of food allergy is at an early stage. Further large-scale studies are needed to elucidate the role of epigenetics in WA-associated immune regulation.

## 3. Clinical Features and Related Disorders

Allergic responses to wheat can manifest in a variety of clinical symptoms, ranging from localized to severe systemic reactions occurring within minutes to hours after wheat exposure [3,6,44,45]. Typical immediate symptoms include gastrointestinal discomfort (nausea, abdominal pain, vomiting, and/or diarrhea), respiratory difficulties (rhinitis, wheezing, and/or asthma), and skin manifestations (eczema, redness, itching, urticaria, and/or angioedema). In severe cases, systemic reactions such as hypotension, hypothermia, or anaphylaxis may also occur; the latter affects multiple organ systems and can rapidly become life-threatening.

Wheat-dependent exercise-induced anaphylaxis (WDEIA), a distinct form of WA presenting as asthma, dyspnea, urticaria, angioedema, syncope, and more rarely shock, is typically diagnosed in adults but occasionally also in older children [46,47,48,49]. WDEIA may occur 10 to 60 min after physical activity when preceded by wheat ingestion 10 min to four hours previous or upon wheat consumption immediately after exercise. Matsuo et al. found that exercise might accentuate allergic reactions in WDEIA by increasing gliadin absorption from the gastrointestinal tract [50], while another Japanese study suggested a role for filaggrin in WDEIA [51]. The severity of clinical manifestations may be dependent on the amount of wheat ingested and exercise intensity. Alcohol and non-steroidal anti-inflammatory drugs such as aspirin have been identified as significant risk factors for WDEIA and were found to trigger episodes even without exercise in a subset of patients [52,53]. 

## 4. Diagnosis

### 4.1. Conventional Diagnostic Strategies

**Figure 2:**
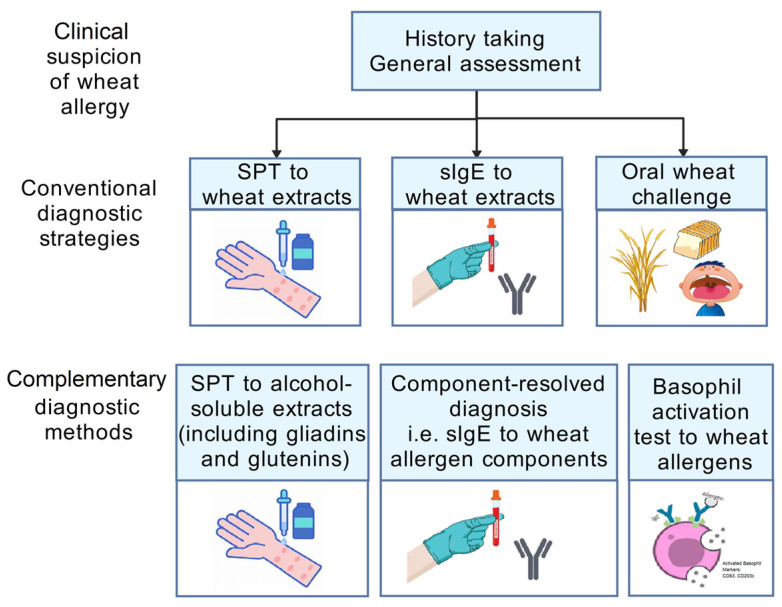
Diagnostic algorithm of wheat allergy. Abbreviations: sIgE, specific immunoglobulin E; SPT, skin prick test. The figure was created with gdp.renlab.cn.

The diagnostic algorithm of WA follows the sequence of history taking, general assessment, and then further investigations (Figure 2). The two routinely used screening tests are a skin prick test (SPT) and a blood test for measuring specific IgE (sIgE) levels, while an oral food challenge (OFC) is performed for confirmation. While the double-blind, placebo-controlled food challenge test (DBPCFC) is considered the gold standard to achieve a definitive diagnosis of WA, an objectively measured open-label OFC may suffice in certain clinical scenarios [54,55,56]. In OFCs, individuals receive incremental doses of whole wheat extracts in a stepwise manner, from one to 50 milligrams (mg) of wheat-specific protein to larger hourly doses, with a cumulative dose of up to 0.5–1 grams (g) of wheat protein [47]. Wheat allergy is diagnosed when (1) the OFC result is positive and (2) symptoms manifest within two hours after ingestion. Although clinical trials have demonstrated the tolerability of wheat allergen-specific OFC, on-site standby of trained healthcare providers may provide subjects with more reassurance in the event of rare near-fatal adverse events. In addition, oral wheat challenge plus cofactors (e.g., exercise or alcohol) could be used to confirm a diagnosis of WDEIA, with the aim of assessing the influence of cofactors on reactivity to wheat allergens [49].

The most commonly adopted diagnostic methods of WA are SPT and sIgE measurement. Although the latter enjoys higher sensitivity, these tests are both of low specificity [5,57]. A postulation for such drawback may be that commercially prepared wheat extracts fail to contain the salt-insoluble major wheat allergens, i.e., gliadins and glutenins [5,57,58,59]. This concern has been addressed by promising wheat extract formulations with gliadin and glutenin-solubilizing properties, which yielded better accuracy for diagnosing WA in children than their preceding commercial wheat extracts [60,61,62]. Another postulation is that extensive allergen cross-reactivity between wheat flour and grass pollen may lower the specificity of SPT and sIgE to wheat. More than 60% of subjects with grass pollen allergy showed false positive results in sIgE to wheat, although they were all clinically asymptomatic [63,64]. Another observation unique to WA is that wheat allergen-specific IgE levels may be falsely elevated in wheat-tolerant individuals—in children who previously had WA but developed tolerance, about 27% to 40% still display elevated levels of sIgE to whole wheat extract (>50 kU/L) in one to two years [65,66]. These factors altogether account for the low specificity of conventional SPT and sIgE measurements.

### 4.2. Wheat Allergens and Component-Resolved Diagnosis 

Wheat belongs to the grass family *Poaceae* and contains numerous allergenic proteins, which can be classified into two main fractions based on solubility in salt [5,45,67]. The salt-soluble fraction, constituting 15–20% of total proteins, includes albumins and globulins containing proteins such as α-Amylase/Trypsin inhibitors (ATIs) and lipid transfer proteins (LTPs). In contrast, the salt-insoluble fraction, comprising approximately 80% of wheat protein content, consists of gliadins and glutenins. Gliadins can be categorized into α/β, γ, and ω-gliadins based on their electrophoretic mobility under acidic conditions. Additionally, ω-gliadins can be further subdivided into ω1, ω2, and ω5 components. Glutenins can be separated via electrophoresis into high molecular weight (HMW) and low molecular weight (LMW) glutenin subunits [5,45,67]. 

In recent years, component-resolved diagnosis (CRD) has been increasingly utilized to identify specific components that can predict clinical reactions in cases of IgE-mediated WA and WDEIA. Commercial tests are now available to measure IgE sensitization to Tri a 19 (ω-5 gliadin) and Tri a 14 (non-specific LTP). Wheat ω-5 gliadin (Tri a 19) is well understood as the major allergen in WDEIA-affected individuals [46,68,69,70]. Studies have also revealed that ω-5 gliadin-specific IgE is a strong predictor of immediate WA in children, with elevated levels correlating with positive results in OFC; ω-5 gliadin-specific IgE was detected in more than 80% of children with WA [55,58,71]. Meanwhile, wheat flour non-specific LTP is an important allergen for IgE-mediated WA, WDEIA, and Baker’s asthma; this protein exhibits resistance to heat and enzymatic degradation, retaining its allergenic potential even after food processing and digestion. Non-specific LTP also does not cross-react with grass pollen; thus, measurement of non-specific LTP may help differentiate wheat sensitization from pollen allergies, which is crucial in patients with high levels of grass pollen-specific IgE [5,44,47]. Furthermore, positive sIgE responses to glutenins, α-, β-, and γ-gliadins, as well as α-Amylase/Trypsin inhibitors have been found in wheat-allergic children, yet none has reached a high specificity and sensitivity in WA diagnosis [57,72,73]. Further studies are required to better understand these wheat allergens.

### 4.3. Cell-Based Diagnosis

An emerging in vitro test to complement WA diagnosis is the basophil activation test (BAT) through the use of fluorescence-based flow cytometry [74,75,76,77]. BAT involves collecting a blood sample and incubating it with specific allergens. Basophils in the sample are identified using surface markers, and their activation is measured by the expression of activation markers like CD63 or CD203c. CD63 and CD203c are upregulated upon basophil degranulation, though CD203c is also expressed in resting basophils [74,78]. Our group showed that the basophil activation test is a sensitive biomarker for the clinical severity of allergic reactions to seawater shrimp [79,80]. Measurement of basophil CD203c expression induced by various preparations of wheat proteins, particularly ω-5 gliadin, is also useful in predicting causative allergens in patients with WA and WDEIA [78,81]. Particular caution is required for subjects to completely avoid wheat intake before BAT as otherwise, subjects’ basophils can be substantially activated at baseline. This problem can influence the accuracy of BAT results that assess the fold changes in CD63 or CD203c expression on basophils from baseline to after in vitro wheat exposure. The mast cell activation test (MAT) is another emerging allergy test that assesses the allergen-specific and dose-dependent responses of mast cells. Under MAT, primary human blood-derived mast cells were generated from circulating precursors. The mast cells are sensitized by mixing with subjects’ serum samples and then incubated with allergens under testing. The final mixture is subjected to flow cytometry to measure the allergen-specific release of mast cell mediators (e.g., β-hexosaminidase) or the expression of surface activation markers (e.g., CD63, CD107a) [54,77,82]. There is at present very limited application of MAT in the clinical setting.

Generally, in the diagnosis of food allergies, SPT and sIgE to extracts exhibit high sensitivity, whereas sIgE to components and the BAT show high specificity. A meta-analysis has shown that the SPT and sIgE are less accurate in diagnosing allergies to sesame, soy, wheat, and shrimp. However, the BAT has demonstrated very high specificity in diagnosing food allergies to peanuts (90%) and sesame (93%) [83] as well as to shrimp (94%) [80]. In addition, the suggested cut-off values for diagnosing WA are 3 mm for SPT, 0.6 kU/L for sIgE to wheat, and 0.3 kU/L for sIgE to ω-5 gliadin [54,83].

## 5. Management 

### 5.1. Natural History of Wheat Allergy

Despite the high prevalence of WA, a considerable proportion of wheat-allergic children develop tolerance later in childhood, showing that WA has a better prognosis than other allergies (e.g., peanuts, shellfish, or fish) which often persist into adulthood. Several studies have shown that the median age of tolerance to wheat allergens is approximately six years of age [65,66]. Resolution rates were found to be 20–29% by four years, 52–56% by eight years, 65–66% by 12 years, and 76% by 18 years. The progress of tolerance can be assessed by measuring wheat allergen-specific sIgE titers repeatedly while on an elimination diet. As the wheat allergen-specific sIgE concentration rises, tolerance development becomes less likely, whereas declining sIgE titers suggest a higher likelihood of tolerance [44]. An American study showed that for children with peak wheat sIgE levels below 20 kU/L, the median age of tolerance development was 2.6 years; for those with peak sIgE levels between 20 and 49 kU/L, it was 4.5 years; and for those with peak sIgE levels ≥ 50 kU/L, it was 12.1 years of age [65]. The abovementioned findings suggest that peak wheat sIgE level is a useful predictor of tolerance development. 

### 5.2. Therapeutic Strategies

The current management approach for IgE-mediated WA primarily involves strict avoidance of wheat-containing foods. Patients need to be educated to accurately identify wheat allergens in food labels and written instructions should be provided to effectively eliminate wheat from their diet [5,7]. According to the European Academy of Allergy and Clinical Immunology (EAACI), the level of avoidance should be tailored to individual symptoms. Those with a history of anaphylaxis should strictly avoid even trace amounts of wheat. For individuals with delayed symptoms and negative IgE tests for wheat, it is advisable to consume the maximum amount that does not trigger symptoms [84]. Individuals with WDEIA should especially refrain from exercising for up to six hours after consuming wheat or wheat-containing products; sometimes, complete wheat avoidance may even be recommended [6,48]. 

However, adhering to a wheat-free diet poses challenges due to the ubiquitous presence of wheat in various food items, including cakes, noodles, pasta, bread, and condiments like soy sauce. Severe accidental exposure to wheat allergens may occur, necessitating the use of epinephrine autoinjectors to manage allergic reactions and subsequent emergency department admission for close monitoring [5]. Antihistamines, glucocorticoids, and β-agonists are considered adjunctive treatments for anaphylaxis which may be used alone or in combination depending on the severity of the reaction [7]. According to EAACI, for individuals with a history of severe reactions during food challenges or following accidental ingestion of wheat-containing products, careful instruction on the use of an adrenaline autoinjector is essential. For those experiencing delayed reactions or mild systemic reactions, administering an age-appropriate dose of an antihistamine is usually sufficient. Due to its rapid absorption, cetirizine is often preferred over other antihistamines such as loratadine or desloratadine [84]. Notably, the Asia–Pacific Research Network for Anaphylaxis found that fewer than two-thirds of anaphylaxis cases received adrenaline treatment. The rate of pre-hospital adrenaline administration was low, and the prescription of adrenaline devices was inadequate across Asia, particularly in developing countries. These findings highlight significant areas for improvement in the current management of anaphylaxis in the region [2].

### 5.3. Immunotherapy

Immunotherapy is a promising method for treating food allergies, with three main routes of administration—oral immunotherapy (OIT), sublingual immunotherapy (SLIT), and epicutaneous immunotherapy (EPIT) [7,85], among which OIT is the most studied. Currently, there are no commercially available products for allergen-specific immunotherapy, and insufficient published data are available to determine the appropriate product or establish adequate protocols [84]. Tomsitz et al. reported that SLIT increased reaction thresholds in three WDEIA-affected patients [86]. However, the feasibility of SLIT in inducing unresponsiveness to wheat can be limited by the daily dosing of wheat that subjects can receive. Oral administration of OIT is the natural way through which subjects are exposed to wheat in their diet, hence the plethora of research data from studies that evaluated the potential usefulness of OIT in treating WA. Oral immunotherapy works by inducing oral tolerance to allergenic foods through modification of both innate and adaptive immune responses and adopts the abovementioned desensitization mechanism. Furthermore, there have not been any reported studies on EPIT for WA.

#### 5.3.1. OIT Protocol

**Figure 3:**
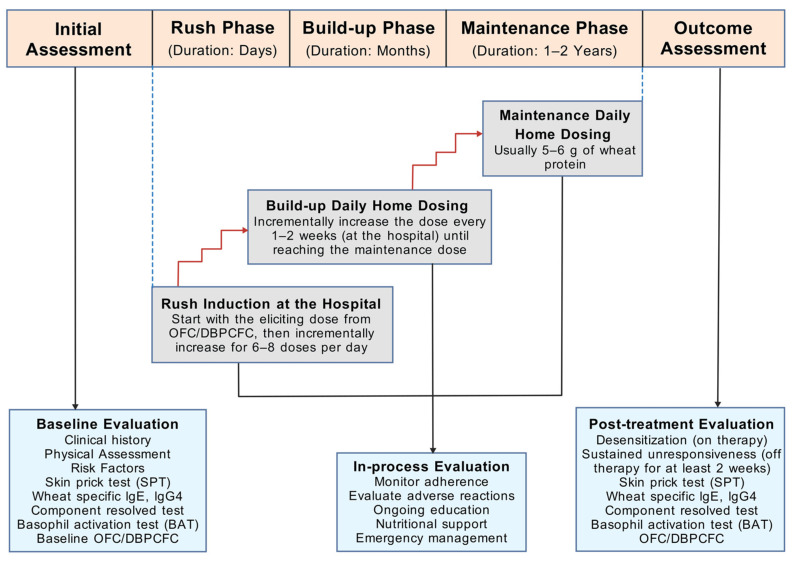
Schematic view of the study design of oral immunotherapy for wheat allergy [9]. Abbreviations: DBPCFC, double-blind, placebo-controlled food challenge; g, gram; IgE, immunoglobulin E; IgG4, immunoglobulin G 4; OFC, oral food challenge. The figure was created with gdp.renlab.cn.

Standard OIT protocols typically comprise the rush, long-term build-up, and maintenance phases (Figure 3) [9]. During the rush phase, patients first consume a very small dose of the test food (equivalent to the eliciting dose from DBPCFC), followed by a gradual increase in the dosage with six to eight doses per day. This phase is usually conducted in a hospital due to the high risk of systemic reactions. Once completed, patients are advised on a safe starting dose for self-administration at home. The subsequent build-up phase involves an incremental increase in immunotherapy dosage every one to two weeks, typically by a 20–30 percent increment per visit, until the maintenance dose (usually one serving dose) is reached. Patients maintain this dose for at least one to two years, followed by evaluation for desensitization and tolerance [also known as sustained unresponsiveness (SU)]. Desensitization indicates subjects’ ability to tolerate the maintenance dose when regularly consuming the food, whereas SU reflects their ability to tolerate doses even after discontinuing regular dosing. Throughout the treatment process, oral food challenge tests are performed to assess desensitization. Subjects with established SU should discontinue the allergenic food doses for at least 2 weeks before undergoing another oral food challenge [9]. The occurrence of SU is the closest surrogate for immunological tolerance as subjects are only intermittently exposed to the food item to which they were previously allergic. Nonetheless, long-term follow-up of subjects for at least 1–2 years is required to ascertain if they have achieved immunological tolerance.

#### 5.3.2. Clinical Trials

Table 1 summarizes the published clinical trials for wheat OIT protocols, demonstrating wide variability, with efficacy being defined differently between trials [20,21,22,59,87,88,89,90,91,92,93,94,95]. For example, the types of wheat products utilized for the immunotherapy varied among the studies—some used pasta whereas others used udon and bread, to name a few. Additionally, the target maintenance doses in different OIT trials ranged from 53 mg to 13 g of wheat protein (WP). Most studies used a maintenance dose of 5–6 g of WP [21,88,89,94], which is approximately equivalent to two to three slices of bread. The varying dosages may have been affected by the degree of wheat sensitization and reports on subjects’ tolerance levels. For instance, in a wheat DBPCFC trial by Rodrigeuz del Rio et al. with higher threshold doses (ranging from 0.8 g to 12.5 g of WP), the subjects were reported to be tolerant even to high maintenance doses of wheat (13 g of WP) without experiencing systemic reactions [87]; this may be attributed to a lower degree of wheat sensitization among this group of patients. Furthermore, maintenance durations varied widely, ranging from two months to three years. Some trials defined their primary endpoint as desensitization to a maintenance dose of OIT, with effectiveness ranging from 30% to 100%. Others defined their primary endpoint as SU to an oral wheat challenge after abstaining from wheat consumption for at least two weeks, with efficiency ranging from 13% to 100%. These factors altogether accounted for the varying degrees of measured efficacy and effectiveness, posing challenges for direct comparison across trials.

Analysis from independent studies suggests that therapeutic outcomes are more dependent on the target dose of wheat protein and the duration of maintenance rather than the type of wheat products used. OIT trials employing higher maintenance doses and longer durations have demonstrated higher rates of desensitization. In a multicenter, randomized controlled trial comparing lower (1445 mg) to a higher dose (2748 mg) OIT, 30.4% versus 57.1% of subjects were able to tolerate a challenge of 7443 mg of WP, respectively [90]. In another study in which children were randomly assigned to the low-dose (650 mg) versus high-dose (2.6 g) OIT groups, 17% and 50% of subjects achieved SU after one year, respectively [91]. In terms of OIT safety, the incidence of total adverse reactions per total ingestion time was significantly lower in the low-dose OIT group compared to the high-dose OIT group (4.76% vs. 8.82%) [91], suggesting that the low-dose protocol may be safer despite lower effectiveness. Furthermore, the duration of treatment may also impact OIT effectiveness. Nagakura et al. reported that during OIT treatment, the proportion of patients achieving short-term unresponsiveness increased by 7%, 28%, and 41% after one, two, and three years, respectively. Moreover, the frequency of adverse reactions per OIT dosing significantly decreased annually [92], indicating that the longer OIT protocol may be more effective and safer. Overall, exploring the maintenance dose and duration of OIT will crucially impact the optimization of the wheat OIT protocol.

Apart from influencing individuals’ clinical response to allergens, the efficacy of wheat OIT is also reflected by changes in SPT and sIgE test results. Several studies, though not all, have reported initial increases and subsequent decreases in sIgE levels to wheat or ω-5 gliadin over the course of OIT [20,21]. Some studies have reported reductions in sIgE levels to wheat or ω-5 gliadin upon completion of the OIT regimen [22,88,91,92,93,94,95]. However, in many cases, individuals still exhibited elevated sIgE levels even upon desensitization or sustained unresponsiveness. Alongside changes in sIgE, a decrease in SPT wheal sizes has been reported in some wheat OIT trials [20,21,89], although this was not observed in several other studies [90,95]. Levels of sIgG4 to wheat and ω-5 gliadin generally increased with wheat OIT [22,87,90], but such a finding was not consistent in other studies [91,95,96]. In summary, OIT may bring about changes in SPT, sIgE, and sIgG4 levels to wheat or ω-5 gliadin in some cases, which can aid in estimating treatment efficacy, although the correlation of these changes with desensitization or SU remains unclear. Apart from SPT and specific Ig levels, the basophil activation test is another possible biomarker of wheat OIT treatment response, yet there has been little research in this area. These diagnostic fields present interesting questions for future research to address.

#### 5.3.3. Precautions for OIT

Exercise should be avoided after wheat ingestion in individuals undergoing OIT, even in those who have achieved short-term SU, since they may be at risk of WDEIA or exhibit exercise-induced allergic reactions upon desensitization (EIARDs). A study evaluating the two-year follow-up prognosis for WA patients who achieved short-term SU after receiving wheat OIT revealed that six out of eight symptomatic patients experienced adverse allergic reactions after exercise and three of them developed anaphylaxis [97]. Studies reported that EIARDs occurred not only after rush OIT but also after slow OIT for wheat, with 66.7% (14/21) and 48.4% (15/31) of WA patients developing EIARDs, respectively [98,99]. Therefore, exercising after wheat intake may be the primary trigger for allergic symptoms during or after OIT.

### 5.4. Other Therapeutic Approaches

The combination of biologics with food OIT is a heated research topic, yet the use of omalizumab which removes circulating IgE antibodies for wheat OIT has not been tested to date. A case report demonstrated that administering omalizumab three weeks before increasing the wheat dose helped achieve higher build-up doses [100]. Chinuki et al. suggested that short-term (12 weeks) omalizumab inhibited wheat allergen-induced basophil activation in hydrolyzed wheat protein-allergy patients, but the effect diminished after treatment cessation [75]. In a long-term (48 weeks) omalizumab study involving 20 adult patients with WDEIA, over 80% of the subjects achieved a basophil activation rate of less than 10% against all fractionated wheat preparations, with 68.8% spared from any allergic reactions [76]. Further studies with larger sample sizes and controlled trials are needed to fully evaluate the efficacy of omalizumab in conjunction with wheat OIT. Furthermore, several probiotics are a feasible treatment option for food allergy via the stimulation of Treg cells [36,42,43]. An Australian multi-center clinical trial reported the safety benefit of a probiotic adjuvant during OIT for peanut allergy, but it did not improve the occurrence of sustained unresponsiveness [101]. There has not been any clinical trial to investigate the therapeutic benefits of a combination of probiotics and wheat OIT. Meanwhile, several strategies have been proposed to improve outcomes in wheat OIT, including wheat modification and adjunct medications such as antihistamines or leukotriene antagonists [5], but the safety and effectiveness profiles of these treatments are yet to be presented. 

## 6. Conclusions and Future Trends

IgE-mediated WA is characterized by immune dysregulation, leading to the release of inflammatory mediators from mast cells and basophils. It can manifest as a spectrum of allergic reactions which impair individuals’ quality of life. For children with IgE-mediated WA, the prognosis is generally positive. The diagnosis of WA involves the commonly used SPT and specific IgE measurement to wheat extracts, yet in view of their low specificity, emerging diagnostic approaches like alcohol-soluble extracts for SPT, component-resolved diagnosis, and the basophil activation test should be combined to enhance the accuracy of WA diagnosis. While the principal management strategy for patients lies in dietary wheat avoidance, oral immunotherapy has recently emerged as a promising approach for treating WA, with trials using higher maintenance doses and extended durations showing increased rates of desensitization. Changes in SPT, sIgE, and sIgG4 levels to wheat or ω-5 gliadin may help assess treatment efficacy, but the inconsistency in currently published trials warrants further confirmation through large-scale studies. There are currently no clinical trials using the BAT—a promising diagnostic method—to evaluate the efficacy of wheat OIT and more research is needed. Lastly, integrating omalizumab as an adjunctive therapy to wheat OIT holds potential for enhancing OIT effectiveness and represents a future direction of research. Further studies are crucial to determine the optimal OIT protocol that would safely promote tolerance in patients with WA.

## Figures and Tables

**Table 1:** Overview of wheat oral immunotherapy trials.

Study	Design	Patients Treated with OIT, n	Age, Mean (Range)	Form of Wheat Use	Up-Dosing Phase	Maintenance Phase	Target Dose	Changes in SPT Scores (Mean/Median)	Changes in sIgE (Mean/Median)	Changes in sIgG (Mean/Median)	Efficacy after OIT, % Desensitization	Efficacy after OIT, % Sustained Unresponsiveness	Adverse Reaction (%)
Rodríguez del Río (2014) [87]	Open-label, nonrandomized, no control	6	5.5 (5–11) years	Semolina porridge and boiled semolina pasta	3–24 days	6 months	13 g of WP	No significant changes but showed a trend (6 mm vs. 2 mm) after 6 months	No significant changes in sIgE to wheat but showed a trend of increase after up-dosing, followed by a decrease after a 6-month follow-up (47.5 vs. 84.55 vs. 28.75 kUA/L)	Increased sIgG4 and sIgG1 to wheat and a panel of wheat proteins in all patients after 6 months	83%	Not assessed	6.25% of doses during up-dosing with none treated with IM epi
Sato (2015) [88]	Open-label, nonrandomized, historical control	18	9.0 (5.9–13.6) years	Boiled udon noodles	5 days	>3 months	5.2 g of WP	Not assessed	Decreased sIgE to wheat (>100 vs. 43.5 kU/L) after 2 years	Not assessed	88.9%	OIT: 61.1% Historical control: 9.1%	26.4% of inpatient doses; 6.8% of outpatient doses with 1 treated with IM epi
Khayatzadeh (2016) [89]	Open-label, nonrandomized non-placebo control	Rush method: n = 8 Outpatient method: n = 5	7 (5.5–19) years	Bread	Rush method: 3–6 days Outpatient method: 66–87 days	3 months	5.2 g of WP	Rush method: decreased (9 mm vs. 6.6 mm) after 3 months; Outpatient method: decreased (9 mm vs. 6.8 mm) after 5 months	Not available	Not assessed	92.3%	Not assessed	Rush method: 29.6% of doses during up-dosing with 5.6% treated with IM epi; Outpatient method: 2.5% of doses during up-dosing with none treated with IM epi
Rekabi (2017) [20]	Open-label, nonrandomized, no control	12	2.25 (2–10) years	Semolina flour and spaghetti (containing pasta)	6.5 months	18 months	70 g of pasta	Decreased (10 mm vs. 3 mm) after 2 years	Decreased total IgE (490 vs. 338.5 IU/mL) after 2 years. sIgE to wheat increased after desensitization, followed by a decrease after the follow-up phase (55.9 vs 65.1 vs. 4.6 IU/mL)	Not assessed	100%	Not assessed	0.06% of doses during up-dosing
Kulmala (2018) [59]	Multicenter, open-label, nonrandomized, no control	100	11.6 (6.1–18.6) years	Boiled wheat spaghetti	4.3 months	12 months	2 g of WP	Not assessed	The three samples available showed decreased sIgE to wheat, gluten, and ω-5 gliadin after OIT	Not assessed	57%	Not assessed	94% of patients; 11 patients used 12 doses of IM epi
Nowak-Węgrzyn (2019) [90]	Multicenter, double-blind, randomized, placebo-control	Low-dose group: n = 23 Placebo group: n = 23 and then crossed over to high dose after 1 year	8.7 (4.2–22.3) years	Vital wheat gluten	11 months	2–14 months	Low dose: 1445 mg of WP High dose: 2748 mg of WP	No significant differences in SPT scores between groups at year 1	No significant differences in sIgE to wheat and ω-5 gliadin between groups at year 1	Increased sIgG4 to wheat and ω-5 gliadin in the OIT group at year 1	Placebo group: 0% after 1 year Low dose: 30.4% after 2 years; High dose: 57.1% after 1 year	Low dose: 13.0% after 2 years	Low dose: 15.4% of doses at year 1 with 0.08% treated with IM epi; 3.1% at year 2 with none treated with IM epi; High dose: 13.4% of doses after 1 year with 0.07% treated with IM epi
Nagakura (2020) [22]	Open-label, nonrandomized, historical control	16	6.7 (5.8–10.7) years	Boiled udon noodles	1 month	11 months	53 mg of WP	Not assessed	Decreased sIgE to wheat (293 vs. 153.5 kUA/L) and ω-5 gliadin (7.5 vs. 4.1 kUA/L) after 1 year	Increased sIgG to wheat (19.8 vs. 24.1 mgA/L) and ω-5 gliadin (6.0 vs. 7.3 mgA/L) after 1 month. Increased sIgG4 to wheat (2.07 vs. 4.7 mgA/L) and ω-5 gliadin (0.07 vs. 0.09 mgA/L) after 1 month	88%	OIT: 69% Historical control: 9%	32.1% of inpatient doses and 4.1% of outpatient doses; none treated with IM epi
Ogura (2020) [91]	Multicenter, open-label, randomized, non-placebo control	Low-dose group: n = 12 High-dose group: n = 12	Low dose group: 5.5 (4.5–5.8) years High dose group: 5.0 (3.7–5.5) years	Boiled udon noodles, boiled pasta, and bread	24 months		Low dose: 650 mg of WP; High dose: 2.6 g of WP	Not assessed	Decreased sIgE to wheat after 1 year in both groups and decreased sIgE to ω-5 gliadin in the low-dose group	No changes in sIgG and sIgG4 to wheat or ω-5 gliadin in both groups	Low-dose group: 66.7%; High-dose group: 33.3% at year 1	Low-dose group: 16.7% at year 1, 58.3% at year 2; High-dose group: 50.0% at year 1, 58.3% at year 2	Low-dose group: 4.76% of doses with 0.02% treated with IM epi; High-dose group: 8.82% of doses with none treated with IM epi
Sugiura (2020) [93]	Open-label, nonrandomized, non-placebo control	35	5 (4–6) years	Boiled udon and somen noodles	12 months		10 times greater than the initial dose	Not assessed	Decreased sIgE to wheat (97.0 vs. 51.9 UA/mL) and ω-5 gliadin (4.8 vs. 1.4 UA/mL) after 12–15 months	Not assessed	OIT: 37.5% Control (wheat avoidance): 10.0%	Not assessed	0.64% of doses with none treated with IM epi
Babaie (2022) [21]	Open-label, nonrandomized, no control	20	6 (2–17) years	Cake and bread	Not mentioned	3–27 months	5.28 g of WP	Decreased (9.8 mm vs. 4.3 mm) after a 3-month maintenance phase	sIgE to wheat increased after up-dosing, followed by a decrease after a 3-month maintenance phase	Not assessed	Not mentioned	47.1% after 3 months, 82.4% after 15 months, and 100% after 27 months	7.2% of doses during up-dosing with 0.4% treated with IM epi
Nagakura (2022) [92]	Open-label, nonrandomized, historical control	29	6.7 (6.3–7.9) years	Boiled udon noodles	1 month	35 months	53 mg of WP	Not assessed	Decreased sIgE to wheat (278 vs. 89.3 kUA/L), gluten (358 vs. 86.9 kUA/L), and ω-5 gliadin (12.7 vs. 3.5 kUA/L) after 3 years	Not assessed	100%	OIT: 7% at year 1, 28% at year 2, and 41% at year 3; Historical control: 0%	7.7% of doses at year 1, 3.9% at year 2, and 2.4% at year 3, and 0.03% treated with IM epi at year 1
Sharafian (2022) [94]	Open-label, nonrandomized, no control	26	6.2 (4–11) years	Bread	6 days	12 months	5.2 g of WP	Not assessed	Decreased sIgE to wheat (90.4 vs. 66.5 IU/mL) after 1 year	Not assessed	100%	93.3%	21.4% of doses; 23.8% of reactions treated with IM epi
Pourvali (2023) [95]	Open-label, nonrandomized, no control	19	6.6 (2.4–16.6) years	Bread and boiled spaghetti	6–7.5 months	7–9 months	5–10 g of WP	No changes after OIT	Decreased sIgE to wheat (108 vs. 24.6 kU/L) after OIT	No changes in sIgG4 to wheat after OIT	68.4%	68.4%	Not mentioned

Abbreviations: g, gram; IM epi, intramuscular epinephrine; mg, milligram; mm, millimeter; OIT, oral immunotherapy; sIgE, specific immunoglobulin E; sIgG, specific immunoglobulin G; SPT, skin prick test; WP, wheat protein.

## Data Availability

Not applicable as this review article does not involve any research data.
